# Development of a Novel Web-Based Intervention Targeting Pain-Related Outcomes in Individuals With Chronic Orofacial Pain: Protocol for a Mixed Methods Study

**DOI:** 10.2196/71839

**Published:** 2025-08-20

**Authors:** Brenda C Lovette, Jafar Bakhshaie, Ronald Kulich, Jeffry R Shaefer, Hsinlin Thomas Cheng, Shuhan He, Ana-Maria Vranceanu, Jonathan Greenberg

**Affiliations:** 1 Center for Health Outcomes and Interdisciplinary Research Department of Psychiatry Massachusetts General Hospital Boston United States; 2 Harvard Medical School Harvard University Boston, MA United States; 3 Department of Anesthesia Critical Care and Pain Medicine Massachusetts General Hospital Boston United States; 4 Oral and Maxillofacial Surgery Department of Surgery Massachusetts General Hospital Boston United States; 5 Department of Neurology Massachusetts General Hospital Boston United States; 6 Lab of Computer Science Massachusetts General Hospital Boston, MA United States

**Keywords:** chronic orofacial pain, mind-body, psychosocial, web-based platform, asynchronous

## Abstract

**Background:**

Chronic orofacial pain (COP) is common, costly, and associated with substantial pain interference and emotional distress. Psychosocial treatments for COP are scarce and limited (eg, rely on talking, which is often painful for this population; require intensive resources, limiting scalability). Here, we describe the study protocol for developing *Face-Forward-Web*, a “talk-free” web-based mind-body intervention for patients with COP.

**Objective:**

We aim to (1) develop *Face-Forward-Web* with the aid of live-video focus groups with adults with COP and (2) optimize *Face-Forward-Web* and our study protocol through beta testing followed by an open feasibility trial.

**Methods:**

We will accomplish these aims in 2 phases, incorporating user-centered design principles. For phase 1, we conducted semistructured focus groups (n=4 groups, 22 participants) with individuals with COP. We are using rapid data analysis followed by thematic analysis to gauge treatment needs, preferences, and perceptions of the proposed platform and skills. This information will inform session structure and content as well as development of a wireframe followed by a prototype. For phase 2, we will conduct beta testing (up to n=10) followed by a feasibility trial (up to n=20) with exit interviews to gather feedback. The primary outcomes are feasibility benchmarks such as recruitment (≥70% of the eligible participants will participate), acceptability (≥70% of the participants complete ≥4 or 5 sessions), credibility, expectancy (≥70% above the Credibility and Expectancy scale’s midpoint), and satisfaction (≥70% above the User Experience Questionnaire’s midpoint).

**Results:**

Recruitment for phase 1 began in October 2024 and concluded in January 2025. Data analysis for phase 1 will conclude in fall 2025 and for phase 2 in 2026. Results will iteratively guide the development of the intervention.

**Conclusions:**

*Face*-*Forward-Web* will be the first talk-free web-based intervention tailored to the needs of adults with COP. Results will inform a future efficacy trial.

**Trial Registration:**

ClinicalTrials.gov NCT06754917; https://clinicaltrials.gov/study/NCT06754917

**International Registered Report Identifier (IRRID):**

DERR1-10.2196/71839

## Introduction

Chronic orofacial pain (COP) is common [1], costly [2], and associated with substantial pain interference (ie, pain-related activity limitations) and emotional distress [3-5]. COP includes a group of conditions that involve pain in the hard and soft tissues of the head, face, and neck that last for more than 3 months [6]. Although the etiological aspects of COP are heterogeneous, COP conditions are unified by a psychosocial profile of substantial maladaptive coping (eg, pain-related fear, pain catastrophizing) [7-9], pain interference, and emotional distress (eg, depression, anxiety) [3,4,10-14], which worsen each other over time [15,16]. Treatments are typically biomedical [4], mostly ineffective [17], and often involve adverse side effects [18], misdiagnoses or incomplete diagnoses [17], and unnecessary/painful procedures (eg, tooth extractions, occlusal adjustment) that may lead to irreversible damage [4,19]. Such treatments do not typically address and often exacerbate activity limitations, maladaptive coping, and emotional distress [4,19]. These psychosocial factors are critical causative elements of COP but are mostly overlooked in COP treatment [20].

Psychosocial interventions such as cognitive behavioral therapy [21-28] and other behavioral programs [29-32] show promise in improving outcomes in individuals with COP [21-32]. However, the existing interventions are limited in several ways: these interventions (1) almost exclusively rely on talking, which is often painful for patients with COP and limits treatment engagement [20,33]; (2) are resource-intensive and require availability of trained providers, limiting accessibility, particularly for underserved communities [34]; (3) often involve costs, travel, and limited insurance coverage [35]; (4) carry a mental health stigma, which limits treatment-seeking and participation [36]; (5) tend to focus on an incomplete diagnosis and mostly temporomandibular disorder, limiting generalizability, and (6) are typically unimodal (eg, cognitive behavioral therapy alone), despite evidence that multimodal interventions work better for complex conditions like COP, which have high psychiatric comorbidity [24,37]. Talk-free asynchronous multimodal web-based platforms can bypass these limitations while maintaining effectiveness [38]. However, to our knowledge, no such interventions exist. Delivering COP interventions through a web-based platform holds potential to expand accessibility, increase scalability, and address this population’s largely unmet needs [20,39].

To this end, we will develop *Face-Forward-Web,* the first web-based platform adapted and refined via mixed methods for COP by using user-centered design principles [40-43]. *Face-Forward-Web* will be adapted from *GetActive*, a mind-body and activity intervention for individuals with chronic musculoskeletal pain [44]. *Face-Forward-Web* will be delivered via a talk-free asynchronous web platform and will address some of the unique and often unaddressed challenges faced by individuals with COP, including difficulty with talking due to pain [20,33], emotional distress [3,5,20], orofacial pain interference and pain intensity [8], and coping with unpredictable pain [20].

**Figure 1:**
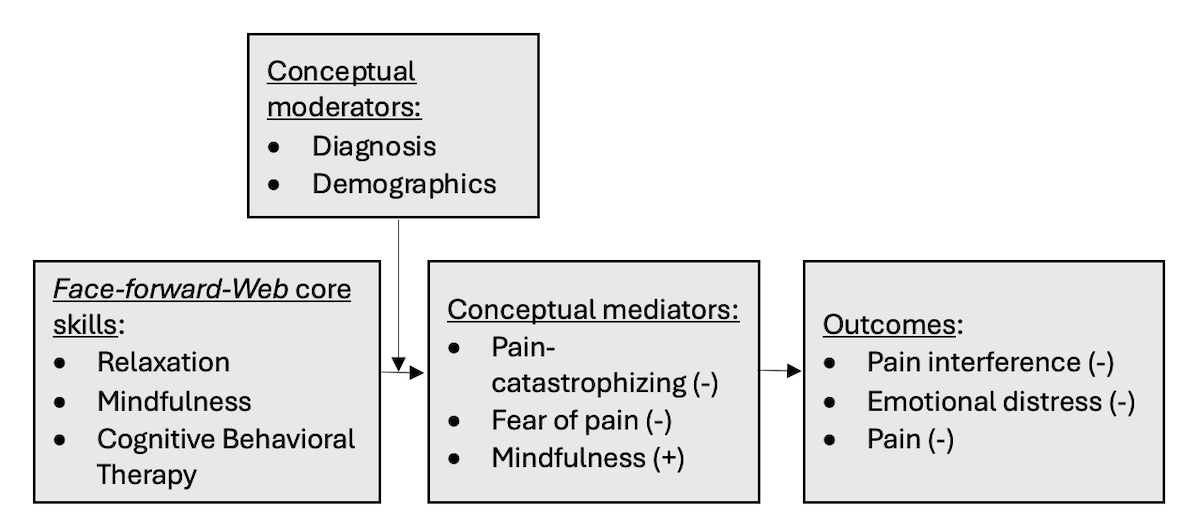
The Face-Forward-Web conceptual model.

Our conceptual model (Figure 1) is informed by the fear-avoidance framework [45,46] and poses that improvement in pain interference, pain, and emotional distress after participation in *Face-Forward-Web* will be explained by improvement in the mechanistic targets of pain catastrophizing, fear of pain, and mindfulness. Our guiding hypothesis (to be tested in future work) is that the *Face-Forward-Web* intervention will be an effective, efficient, and scalable strategy to address the unmet needs and challenges of pain interference and distress among patients with COP.

This study aims to (1) develop *Face-Forward-Web* with the aid of live video focus groups with adults with COP and (2) optimize *Face-Forward-Web* and our study protocol through beta testing (n=up to 10) to refine the intervention followed by a feasibility trial (n=up to 20). The results will inform future efficacy research, evaluating *Face-Forward-Web* versus an active control web platform in improving pain interference, depression, and anxiety.

## Methods

### Study Design and Setting

This study protocol adheres to the SPIRIT (Standard Protocol Items: Recommendations for Interventional Trials) 2013 statement (Multimedia Appendix 1). Our approach is consistent with the National Institutes of Health (NIH) stage model for behavioral intervention development [47,48]. The proposed phases address stages 1A (intervention development and refinement) and 1B (feasibility testing). For phase 2, the primary outcomes are feasibility benchmarks (ie, feasibility of recruitment, credibility and expectancy, acceptability of treatment, adherence to homework, satisfaction [user experience], feasibility of assessments, adverse events).

This study will be completed in 2 main phases conducted at a large academic hospital. In phase 1, we conducted 4 virtual focus groups (22 patients with COP) at which point we reached saturation [49]. We aimed to understand their treatment needs and preferences, perceptions, and recommendations for a web platform delivery, and the best ways to engage and retain participants. We also gathered participants’ feedback on program content via sample videos similar to those intended to be used in the *Face-Forward-Web* program. We are using this information to inform adaptations from the *GetActive* intervention for chronic musculoskeletal pain intervention to the *Face-Forward-Web* intervention for chronic orofacial pain as well as for the development of a wireframe for *Face-Forward-Web*, followed by a prototype [42].

In phase 2, we will conduct beta testing (up to n=10) and revise *Face-Forward-Web* and the study procedures according to the feedback and information gathered. We will then conduct a feasibility trial (up to n=20) with rapid iterative development of the prototype intervention [50].

### Inclusion and Exclusion Criteria

We plan to include participants aged ≥18 years, with English fluency and literacy, chronic nonmalignant COP (>3 months), who express ability and willingness to participate in a live-video focus group/web platform intervention, are free of concurrent psychotropic medications for at least 2 weeks prior to treatment, or stable on current psychotropic medication for ≥6 weeks, and have pain intensity ≥4/10. We will exclude individuals who practiced mindfulness >45 min/wk in the past 3 months and those who have participated in mind-body or cognitive behavioral therapy in the past 3 months; have severe untreated mental health (eg, psychosis) or active suicidal ideation; or are pregnant (per our institutional review board).

### Recruitment

For both phases of the study, we will recruit from sources such as the Facial Pain Association—a large organization providing support, education, and advocacy for individuals with COP—the Facial Pain Clinic at Massachusetts General Hospital, social media, and other sources. We will prioritize recruitment of racial and ethnic minorities and aim to reflect the US population (38% of the minorities). Individuals who participate in phase 1 focus groups may participate in phase 2 feasibility testing.

### Procedures

#### Ethical Considerations

Methodology, including interview script and recruitment strategies and materials, were approved by the institutional review board of the Massachusetts General Hospital. The institutional review board determined that phase 1 activities were exempt from written informed consent. Instead, all participants viewed an online study fact sheet during enrollment and provided consent to participate (protocol 2024P001309). Participants in phase 2 will provide written informed consent and may opt out at any time during the study (protocol 2024P003501). All focus group and exit interview transcripts are deidentified prior to analysis, and any quantitative data exported for analysis will also be deidentified. Participants can receive payment up to US $50 via check for completing all phase 1 activities and up to US $170 for completing all phase 2 activities.

#### Phase 1: Developmental Phase With Focus Group Feedback

We have developed a semistructured qualitative interview script with items assessing treatment needs, preferences, and perceptions of the intervention skills and structure (Multimedia Appendix 2). We completed recruitment and data collection for phase 1 in January 2025. We recruited individuals with COP. Study staff screened potential participants and obtained verbal consent. Then, eligible patients were invited to participate in one of the 4 focus groups. Upon enrollment, participants completed an electronic demographic questionnaire and were aided with downloading and installing the video software (Zoom) as needed. All qualitative interviews were audio-recorded, transcribed, organized, and analyzed using the NVivo 12 qualitative statistical package (QSR International). We will use the qualitative results of phase 1 to develop *Face-Forward-Web* and the treatment manual to be piloted in phase 2.

#### Phase 2: Beta Testing and Feasibility Trial With Exit Interviews

##### Beta Testing

After screening, consent, and enrollment, up to 10 individuals with COP will beta test *the Face-Forward-Web* intervention. The research coordinator will schedule an orientation session, in which she will outline how to navigate the *Face-Forward-Web* platform. Participants will then independently complete the intervention. After each module, participants will be asked to indicate their level of satisfaction from the module on a 1-4 Likert scale and provide feedback on their impressions from the module in a textbox. At the conclusion of the intervention, participants will participate in exit interviews over Zoom. Exit interviews will last ~30 minutes and will be audio-recorded and transcribed. Similar to phase 1, the exit interviews will involve a semistructured script, followed by rapid data analysis and qualitative thematic analysis using NVivo 12. We will utilize results to further refine the intervention’s content manual and procedures as well as refine *Face-Forward-Web* prior to the feasibility trial.

##### Feasibility Testing

Participants will be recruited, screened, and enrolled using the methods described above. After consenting, participants (n=up to 20) will complete a set of valid and reliable online questionnaires over the secure Research Electronic Data Capture (REDCap) system. The estimated time for completion is 20 minutes. If participants miss an item, they will be prompted to complete it, thereby minimizing missing data. Participants will complete the questionaries at 2 additional timepoints—after completing the intervention and again 3 months later. After baseline assessments, participants will participate in an orientation session. Then they will complete the intervention and subsequent exit interviews following similar procedures as in the beta testing.

### The Face-Forward-Web Intervention

*Face-Forward-Web* consists of 5 sessions (Table 1), which teach relaxation (eg, body scan, deep breathing), mindfulness, and cognitive-behavioral skills (eg, adaptive thinking, reducing negative affective reactivity). The content is consistent with *GetActive* [44] and adapted to the unique needs of people with COP and for delivery via the web platform. Due to the individual/asynchronous format of *Face-Forward-Web*, we were able to condense the *GetActive* content into 5 sessions, which may maximize feasibility and engagement (to be tested in the proposed studies). Each session consists of a combination of short (~5-10 minutes) videos conveying educational content and skill delivery. Each session ends with an interactive quiz covering the session’s content to gauge comprehension and facilitate material absorption, which can be an effective intervention element for people with chronic pain [51]. Audio and video clips will be available to guide participants through their daily home practice. Once invited by a research assistant, participants will log in and access the first session’s materials. Contingent on completion of the interactive quiz at the end of each session, access to the subsequent session will be granted in the following week. Participants will be free to review and practice all the skills and materials from the completed sessions until the 3-month follow-up assessment and will be encouraged to use the platform for daily practice. The platform will be easily accessible across multiple interfaces such as a website and mobile app through any internet-enabled device (eg, smartphone, tablet, computer). We will track participants’ homework completion and platform activity. To encourage retention, we will text reminders about the sessions and home practice through the Health Insurance Portability and Accountability Act–compliant texting service, as previously successfully implemented by our group and deemed useful by participants [52].

**Table 1:** Face-Forward-Web session topics and examples of content.

Session	*Face-Forward-Web* topic	Skills and examples of content/videos
1	Taking charge of orofacial pain	Orofacial “Pain alarm” (reactions to COP^a^) Debunking myths about COP Downward and upward spirals (COP is associated with thoughts, feelings, and behaviors that can be maladaptive or adaptive) Deep breathing
2	Managing emotional challenges in orofacial pain	Mindfulness Body scan
3	Working with negative thoughts	Identifying NATs^b^ Checking NATs Challenging NATs
4	Not letting orofacial pain get the best of us	Acceptance Using Face-Forward-Web skills to reengage in activities (eg, daily, recreational, social, family, work-related)
5	Facing forward: “Rolling” with orofacial pain and staying resilient	Mindfulness of discomfort and pain Review all skills Create personalized plan

^a^COP: chronic orofacial pain.

^b^NAT: negative automatic thought.

### Treatment Delivery and Protocol Adherence

Participants will continue their usual care as determined by their medical providers (eg, medication, occlusal splits and adjustments, physical therapy). We will determine fidelity to *Face-Forward-Web* via a user experience software such as Google Analytics [53]. This will enable us to track participants’ frequency and time spent on each of the web platform’s pages and clicks on exercises and quizzes and evaluate participation, even on partially completed sessions, thereby increasing the accuracy of attendance evaluation.

### Assessments

#### Pain Severity and Pain Interference

The Graded Chronic Pain Scale [54] is a 7-item scale measuring pain severity and pain interference. The time frame of reference will be the past week, consistent with prior work [55,56]. The Numerical Rating Scale [57] is a single item measure assessing pain severity.

#### Anxiety

The Generalized Anxiety Disorder Scale [58] is a 7-item scale that assesses the severity of the anxiety symptoms over the past 2 weeks.

#### Depression

The Patient Health Questionnaire [59] is a 9-item measure that assesses depression symptoms over the past 2 weeks.

#### Catastrophic Thinking About Pain

The Pain Catastrophizing Scale [60] is a 13-item measure assessing hopelessness, helplessness, and rumination about pain.

#### Fear of Pain Due To Movement

The Tampa Scale for Kinesiophobia for Temporomandibular Disorders [61] is a 12-item scale assessing fear of pain due to facial movement and adapted to heterogeneous orofacial pain [62] (ie, expanding references from “jaw” to “jaw or face”)

#### Mindfulness

The Cognitive and Affective Mindfulness Scale-Revised [63] is a 12-item questionnaire that measures an individual's level of mindfulness, including present-focused attention, awareness of thoughts and feelings, and nonjudgmental acceptance of internal experiences. The Applied Mindfulness Process Scale [64] is a 15-item questionnaire that measures respondents’ application of mindfulness practices in daily life.

#### Treatment Satisfaction

The User Experience Questionnaire assesses users’ satisfaction and experience with digital products, software, and platforms [65]. This assessment is administered at 1 timepoint, immediately after the intervention.

#### Treatment Credibility

The Credibility and Expectancy Questionnaire assesses how believable and logical patients perceive the treatment to be [66]. This assessment is administered at 1 timepoint, immediately after the intervention.

### Data Analysis

#### Phase 1: Focus Groups

We used rapid data analysis during focus groups to enable timely application of key findings to iterative refinement of our prototype and procedures. Next, we will use thematic analysis following a hybrid deductive-inductive approach [67,68] for a deeper analysis to gauge treatment needs, preferences, and perceptions of the intervention. We will analyze qualitative data from the interviews by using NVivo 12, which enables reliability assessment (κ) of theme-coding between independent coders. Coders will discuss and resolve potential discrepancies until they reach agreement and sufficient reliability, indicated by κ>.80. We will use the results to make specific adaptations to the program content and procedures based on focus group data to best meet participants’ needs and challenges.

#### Phase 2: Beta and Feasibility Testing With Exit Interviews

We will use frequency and proportions to assess the feasibility of the recruitment and retention procedures. We will determine treatment satisfaction and credibility by the proportion of participants who score above the scale midpoint in the User Experience Questionnaire [65] and Credibility and Expectancy Questionnaire [66], respectively. See Table 2 for further details on the feasibility, credibility, and acceptability benchmarks, consistent with other established feasibility work [67-69]. We will analyze qualitative data from the exit interviews by using the same procedures described in phase 1.

**Table 2:** Feasibility benchmarks.

Outcome	Acceptable	Excellent
Credibility and expectancy	>70% of the participants with score over scale midpoint.	>75% of the participants with score over scale midpoint.
User experience	>70% of the participants with score over scale midpoint.	>75% of the participants with score over scale midpoint.
Feasibility of recruitment	>70% of eligible participants agree to participate.	At least 80% of eligible participants agree to participate.
Acceptability of treatment	>70% of the participants attend 3 out of 5 sessions.	>80% of the participants attend 3 out of 5 sessions.
Adherence to homework	>70% of the participants practice >1 skill on 3 d/wk.	>80% of participants practice >1 skill on 3 d/wk.
Feasibility of assessments	>70% of the participants have no measures fully missing.	>90% of the participants have no measures fully missing.
Adverse events	Minimal	None

## Results

This study is funded by the National Institute of Dental and Craniofacial Research grant 1R21DE033502-01A1 to JG and AMV. Recruitment began in October 2024 for phase 1. Data collection for phase 1 was completed in March 2025. Primary data analysis for phase 1 is expected to conclude by fall 2025 and in 2026 for phase 2. As of August 2025, we have completed 4 planned focus groups with a total of 22 participants representing a variety of COP diagnoses, including temporomandibular joint dysfunction, trigeminal neuralgia, and burning mouth syndrome. The rapid data analysis is complete, and we are beginning thematic analysis. We are starting to create intervention content, informed by results from our focus groups. Once available, we will share the data and results in the Vivli global clinical research data sharing platform.

## Discussion

Pain interference and emotional distress are prevalent among people with COP [62] but are commonly neglected in current treatments [20,39]. Available psychosocial interventions such as cognitive behavioral therapy [21-28] and other behavioral programs [29-32] show promise in improving outcomes in individuals with COP [21-32] but are often not suitable for people with COP due to several limitations. Most notably, these interventions typically rely on verbal communication, which is often painful for this population due to facial pain symptoms, which can be exacerbated by talking. Additionally, such interventions can be associated with stigma and require extensive resources (eg, availability of a trained live clinician). Here, we describe a protocol to develop, test, and refine *Face-Forward-Web*, the first talk-free web-based intervention for adults with heterogeneous COP, tailored specifically to meet their needs and challenges. By teaching mind-body (eg, mindfulness, relaxation) and cognitive behavioral skills within a talk-free modality, this intervention holds potential to bypass barriers and address these commonly neglected needs [38].

We anticipate that the results of phase 1 will align with our preliminary work underscoring the need for a COP intervention that addresses psychosocial factors [20] and uses a web-based platform. We also anticipate findings to highlight specific elements of the intervention’s content, structure, and study procedures that can be tailored to maximize fit for this population’s needs. Assuming the successful integration of these findings, we hypothesize that phase 2 will demonstrate high levels of feasibility across domains (eg, user experience, acceptability, adherence) and no adverse events related to study participation.

We follow the NIH stage model [48] and prioritize establishing feasibility and iterative refinement of the intervention prior to efficacy testing. By integrating qualitative methods, our approach centers on participant needs and preferences, ensuring that the intervention is best positioned to support those impacted by COP. Once both phases are complete, these results will inform a future efficacy trial of *Face-Forward-Web* versus an active control platform.

Potential limitations should be considered. First, the study relies on participants’ self-reported diagnoses and symptoms. This limitation is partially mitigated by our recruitment methods, which include referrals from COP medical specialists and established advocacy groups. Second, our broad diagnostic inclusion criteria may reduce the specificity of the findings. However, our preliminary research suggests that individuals across diagnostic categories share more common needs than differences. This inclusive approach enhances the potential for generalizability to a broader population and may be considered a potential strength. Other strengths include the use of established feasibility benchmarks, validated measures, and following the NIH model for behavioral intervention development.

Our work has yielded a few lessons learned thus far. First, given our small budget, we have had to carefully prioritize spending and have chosen to focus on the remuneration of participants [70]. Additionally, for recruitment, we have found both professional networks (providers who treat COP) and community resources (Facial Pain Association) to be effective. However, meeting our goal of including the perspectives of diverse populations, including a variety of COP diagnoses, as well as racial, ethnic, and medically underserved areas [71] has required additional strategies such as direct engagement of referral providers.

In summary, this protocol describes the development, refinement, and feasibility testing of *Face-Forward-Web*, the first talk-free web platform for adults with heterogeneous COP, tailored specifically to their needs and challenges by using mixed methods. We will utilize the NIH stage model for behavioral intervention development [47,48], the Science of Behavioral Change framework [72], and a user-centered design process to maximize the intervention’s fit for this population’s needs [40,41]. Our results will establish feasibility and directly inform a subsequent efficacy trial.

## Appendix

Multimedia Appendix 1 SPIRIT (Standard Protocol Items: Recommendations for Interventional Trials) checklist.

Multimedia Appendix 2 Focus group interview script.

Multimedia Appendix 3 Peer review report by BMHO - Biobehavioral Medicine and Health Outcomes Study Section Risk, Prevention and Health Behavior Integrated Review Group (National Institutes of Health, USA).

## Abbreviations

COP: chronic orofacial pain
NIH: National Institutes of Health
REDCap: Research Electronic Data Capture
SPIRIT: Standard Protocol Items: Recommendations for Interventional Trials
