# Supplementary material for: Development of a Novel Web-Based Intervention Targeting Pain-Related Outcomes in Individuals With Chronic Orofacial Pain: Protocol for a Mixed Methods Study
Source: JMIR Res Protoc. 2025 Aug 20;14:e71839. doi: 10.2196/71839 (PMC12409173; doi:10.2196/71839)
Supplement: Multimedia Appendix 2 [file resprot_v14i1e71839_app2.docx]

**Understanding treatment needs and preferences for individuals with chronic orofacial pain**

(Focus Group Script)

**Date: ___ / ___ / ______ Leaders: _______________**

**Group number: _______________ Attendance: _______________**

**Facilitator Notes:**

1. Follow prompts in script regarding starting and stopping audio recording.
2. Present every main question, as time allows; notes/highlights included through script indicate questions to prioritize.
3. All probes are optional – present probes only for clarification, when information is not being generated by focus group participants, or when time allows dwelling upon the particular question.
4. Focus group should not exceed 60 minutes.
5. Exit interviews are optional and should not exceed 15 minutes.

**Introduction 0:03**

Thank you for joining us today. My name is _____________________ and I am__________________________. I am part of a multidisciplinary team at Massachusetts General Hospital, interested in developing and testing a program to improve physical and emotional functioning in adults with chronic facial pain. We would like to learn about your experiences with your chronic facial pain and treatment so that our program can be most helpful to other patients like yourself.

A few logistical things:

1. We will talk for about 60 minutes.
2. You are free to leave at any time.
3. You don’t have to answer any questions that might make you feel uncomfortable.
4. It is OK to disagree with us or with other participants – the goal is to get many different opinions to help us develop a program that helps as many patients as possible.
5. We will audio record our conversation so that we can remember everything you say.
6. Your information is confidential, and we ask you not to share this information outside of this group.
7. We ask that you raise your hand before speaking to help us facilitate participation from everyone.
8. If you prefer that we don’t call on you by name, please let us know by raising your hand.

Does anyone have any questions before we begin? Lastly, is everyone comfortable with this session being audio recorded?

*[START RECORDING]*

**Warm Up 0:05**

To start, we would love for everyone to introduce themselves with their first name and something that you enjoy doing.

**Domain 2: Perceptions of *Face-Forward-Web* content, structure and skills 0:22**

***Theme 1: General Perceptions/Impressions of the Program***

We are in the process of developing a web-based program to help individuals with chronic orofacial pain. The program will be self-guided and will include evidence-based skills, videos and information aiming to reduce emotional distress and help participants better manage and cope with their orofacial pain.

What do you think about such a web-based program?

What specific topics you would like to learn about during this program?

***Theme 2: Modality and structure* 0:29**

What are your thoughts about having this program delivered via a self-guided web-platform as opposed to an intervention with a live clinician?
 PROBE: Benefits and drawbacks of web-based platform

Probe: How do you feel about your ability to quickly learn how to use a new website like this?

Probe: What type of support do you think you would need to facilitate your using the website?

The program will include 5 modules or sessions of 45-60 minutes each. Each week the participant would be able to access the next module if they completed the previous one.

What do you think about this structure?
What to you think about the number of modules/sessions, to be completed independently at your own time?

***Theme 3: Perception of skills and content* 0:34**

The Web-Based Platform will teach different types of skills, through videos and experiential practice.

*Mind Body Skills*

We plan to teach mind-body skills, including meditation, to reduce stress help better manage chronic orofacial pain. One of these skills is deep breathing. We will now show you a brief video, ask you to follow the instructions in the video, and will then ask you what you think about it.

[PLAY DEEP BREATHING VIDEO]

What are your thoughts about this?

How relevant or helpful do you think this might be for helping manage some of the challenges you experience as part of your chronic orofacial pain?

Have you tried mind-body skills before? What were your impressions?

Is there anything we can change or improve for the video to be more engaging or effective?

IF TIME ALLOWS:

We will also teach a skill called *Self Compassion*, which aims to reduce self-criticism and teach participants to be less harsh on themselves.

What are your thoughts about this? Have you tried this before?

*Cognitive Behavioral skills* 0:43

We will also help patients learn about how their thoughts, emotions and pain are connected, and help them spot the ways negative thoughts can make pain worse. Let’s now watch a video about this together.

[PLAY DOWNWARD SPIRAL VIDEO]

What are your thoughts about this?

Is there anything we can change or improve for the video to be more engaging or effective?

IF TIME ALLOWS:

[PLAY CATCH CHECK AND CHANGE VIDEO]

What are your thoughts about this?

How relevant or helpful do you think this might be for helping manage some of the challenges you experience as part of your chronic orofacial pain?

Have you tried something like this before? What were your impressions?

Is there anything we can change or improve for the video to be more engaging or effective?

*Quizzes.* We are planning for each session to end with a “quiz” about the session’s content. This is what it may look like
[SHOW QUIZ EXAMPLE]

What are your thoughts about this?
To what degree do you think such a quiz can be helpful in promoting engagement and understanding of the material?
What else can we do to help improve engagement and understanding of session material?

**Domain 3: Barriers/Facilitators to Participation/Adherence** 0:50

***Theme 1: General Barriers/Facilitators***

What might prevent you from independently participating in and completing all 8 modules of a program like this?

Probe: Distractions (e.g., phone/email/social media notifications in the middle of sessions, findings the time? Tech. Literacy? (Lack of) interest?

What would make it easier for you to engage in and complete all sessions/modules of the program?

Probe: Reminders? Calls / follow ups from study staff?

***Theme 2: Barriers/Facilitators to Homework*** 0:56

In order to gain the most out of the program it helps to regularly practice the skills that are taught. One way to sustain practice is to keep track of what you do. In this program, we will ask you to practice the skills we teach for about 10 minutes daily, as well as log your practice.

How do you feel about being asked to complete home practice of the skills learned?

What may get in the way of doing home practice approximately 10 minutes a day for the duration of the 8-week web-based program?

Probe: Time/Availability? Motivation? Distraction?

Is there anything you can think of that would facilitate such home practice?

Probe: Reminders? Specific home practice plan/assignments/routine? More information of value of daily practice? Social support/accountability?

**Domain 1: Perceived Effects of COP and past treatments 0:11**

***Theme 1: impact of COP (NOTE: REVIEW THIS BRIEFLY)***

What aspects of your life have been impacted by your orofacial pain?

- Social / relationships
- Talking
- Psychological – Stress/mood etc.
- Physical function / activities of daily life including talking
- work participation / finances

***Theme 2: Treatment(s)* 0:14**

What treatments have you tried (if any)?

Probe: Have you tried any nonprescribed treatments or pain remedies?

Probe: Any psychological treatment?

What treatments have been most/least effective?

Probe: Did the effects of the treatment(s) worsen/better your quality of life?

**WRAP-UP**

Is there anything else that we did not ask that you would like to share?

*[STOP RECORDING]*

Thank you for your participation in this focus group today! The information you’ve shared with us is helpful and will aid in meeting the needs of individuals with chronic orofacial pain.
