# Supplementary material for: Development of a Novel Web-Based Intervention Targeting Pain-Related Outcomes in Individuals With Chronic Orofacial Pain: Protocol for a Mixed Methods Study
Source: JMIR Res Protoc. 2025 Aug 20;14:e71839. doi: 10.2196/71839 (PMC12409173; doi:10.2196/71839)
Supplement: Multimedia Appendix 3 [file resprot_v14i1e71839_app3.pdf]

SUMMARY STATEMENT

(Privileged Communication)

PROGRAM CONTACT:
LORENA Baccaglini
301-435-7908
lorena.baccaglini@nih.gov

Release Date: 02/28/2024
Revised Date:

Application Number: 1R21DE033502-01A1
Formerly: 1R21DE033502-01

Principal Investigators (Listed Alphabetically):

GREENBERG, JONATHAN (Contact)
VRANCEANU, ANA-MARIA

Applicant Organization: MASSACHUSETTS GENERAL HOSPITAL

Review Group: BMHO
Biobehavioral Medicine and Health Outcomes Study Section

Meeting Date: 01/29/2024
Council: MAY 2024
Requested Start: 07/01/2024

Opportunity Number: PA-20-195
PCC: P4K2

Project Title: Development of "Face-Forward-Web": A novel web-based program targeting pain-related outcomes among patients with heterogeneous chronic orofacial pain
SRG Action: Impact Score:23
Next Steps: Visit https://grants.nih.gov/grants/next_steps.htm
Human Subjects: 30-Human subjects involved - Certified, no SRG concerns
Animal Subjects: 10-No live vertebrate animals involved for competing appl.
Gender: 1A-Both genders, scientifically acceptable
Minority: 1A-Minorities and non-minorities, scientifically acceptable
Age: 1A-Children, Adults, Older Adults, scientifically acceptable

Project Year	Direct Costs Requested	Estimated Total Cost
1	150,000	250,500
2	125,000	208,750
TOTAL	275,000	459,250

ADMINISTRATIVE BUDGET NOTE: The budget shown is the requested budget and has not been adjusted to reflect any recommendations made by reviewers. If an award is planned, the costs will be calculated by Institute grants management staff based on the recommendations outlined below in the COMMITTEE BUDGET RECOMMENDATIONS section.

1R21DE033502-01A1 Greenberg, Jonathan

RESUME AND SUMMARY OF DISCUSSION: This application proposes to adapt a multimodal mind-body intervention to a web-platform to reduce stress and enhance resiliency for adults with chronic orofacial pain (COP) and evaluate its feasibility and acceptability. During discussion, the panel agreed the significance of developing a web-based intervention that reduces face-to-face communication and avoids other current limitations of treatment for COP patients is high, with potential to advance clinical management of pain. These productive multiple-PIs and strong investigative team were responsive to prior review and the application is improved, notably by expanding the literature review of web-based interventions, clarifying methodology, addressing unequal group sizes and providing a recruitment/retention plan. Reviewers noted using an asynchronous web-based platform for delivery of a talk-free psychosocial intervention to reduce barriers to engagement for adults with COP is innovative. Additional strengths identified include preliminary data supporting feasibility and preliminary efficacy, use of NIH model of behavioral iterative intervention development, validated measures and feasibility benchmarks, assessment of intervention satisfaction and engagement and fidelity monitoring. Reviewers also identified addressable weaknesses including limited characterization of sample, insufficient sample size to reduce error and noise, and primarily qualitative analyses with few quantitative benchmarks. Overall, the panel agreed the application's strengths outweighed its weaknesses and will have a high impact advancing clinical management of pain for patients with chronic orofacial pain.

DESCRIPTION (provided by applicant): Chronic orofacial pain (COP) conditions are common, costly, and debilitating. These conditions are unified by high levels of pain-related activity limitations, maladaptive coping and emotional distress, which are most often unaddressed and worsen each other over time. The majority of COP treatments are biomedical, often ineffective, involve adverse side-effects, misdiagnoses, and can entail unnecessary and painful procedures that may lead to irreversible damage. Available psychosocial interventions are limited in effectiveness, accessibility, scalability, scope, availability of trained providers, and insurance coverage. They also rely on talking, posing a significant barrier to participation for many patients with COP. "Talk-free" psychosocial web-based platforms are needed to bypass these limitations and make psychosocial treatment accessible, scalable, and generalizable but do not exist for adults with heterogenous COP. The goal of this R21 proposal is to adapt the Relaxation Response Resiliency Program (3RP), an evidence-based program, for the unique needs of people with COP and for asynchronous delivery through a web-based platform. We have shown that the 3RP improves pain-related activity limitations and emotional distress among people with refractory temporomandibular joint disorder (TMD) when delivered in person. However, to assure accessibility, generalizability, scalability, and future efficacy, it is essential to adapt the 3RP to a "talk-free" web platform tailored to unique unmet challenges in COP. Our guiding hypothesis (to be tested in future work) is that Face-Forward-Web will be an effective, efficient and scalable strategy to improve pain-related activity limitations and emotional distress among patients with COP. Specific Aims and Research Design. We will conduct live video focus groups (N=4-5 groups/ N=20 individuals) with people who have heterogeneous COP to understand treatments needs and preferences and adapt the intervention for asynchronous delivery via a web-based platform - Face-Forward-Web, the first of its kind for this population (aim 1); and (2) iteratively optimize Face-Forward-Web through a series of open pilots with exit interviews (2 with N=5 and 1 with N=10) until critical Go/No-go feasibility and acceptability benchmarks are met (aim 2). This project aligns with NIDCR's stated priorities of supporting research promoting craniofacial health, alleviating discomfort, and embracing technology as a driver of discovery. Impact: Our interdisciplinary team of psychologists, a dentist and a neurologist has partnered with the national Facial Pain Association to collect preliminary data and facilitate recruitment for this project. We have extensive expertise with mixed methods, development of web platforms, and remote clinical trials. Results of this study will directly inform a subsequent UG3/UH3 NIDCR application. In the UG3 we will conduct a small pilot RCT (NIH 1B) to ensure our ability to

randomize and finalize our study protocol, including fidelity measurement. In the UH3 phase we will test the efficacy of Face-Forward- Web in improving pain, activity limitations and emotional distress, and test theory-driven mechanisms of improvement through adaptive coping. Face-Forward-Web, adapted specifically for the needs of this population and delivered through their preferred modality, has the potential to improve pain, activity limitations, and emotional distress in this population.

PUBLIC HEALTH RELEVANCE: Chronic orofacial pain conditions are unified by high levels of pain-related disability, maladaptive coping (e.g., fear avoidance and catastrophic thinking about pain) and emotional distress (e.g., depression and anxiety). The proposed study will develop, iteratively refine, and establish the feasibility, acceptability and credibility of Face-Forward-Web, the first psychosocial intervention developed through mixed-methods, designed specifically for adults with heterogeneous chronic orofacial pain, and delivered through web-platform (this population's preferred treatment modality). Face-Forward-Web bypasses key accessibility and scalability-related limitations of existing interventions (including challenges with talk- based therapy, which is often painful and limits engagement in this population) and has the potential to dramatically improve pain, pain-related activity limitations and distress in this population.

CRITIQUE 1

Significance: 2
Investigator(s): 1
Innovation: 2
Approach: 3
Environment: 1

Overall Impact: This R21 resubmission application from MPIs Greenberg and Vranceanu proposes to develop and establish the feasibility, acceptability, and credibility of a multimodal psychological intervention for adults with chronic orofacial pain. The intervention will be delivered via a web-platform and occur across two phases including a focus group and an iterative pilot study with 20 participants with chronic orofacial pain. The MPIs were largely responsive to previous critiques. Goals of assessing feasibility and acceptability, and making iterative changes to the treatment manual, are appropriate for this early stage of research in this area. The investigators are strong, and the environment is excellent. Notable strengths of the proposal include an MPI team with expertise in clinical trials methodology, innovative aspects of the intervention design (targeting concepts of resilience), and strong preliminary data to support the feasibility of the study. Some weaknesses raise concerns, including the heterogeneity of the study sample, thresholds for inclusion, and lack of detail on and confirmation of included diagnoses. Given the strengths and weaknesses in the approach, the potential overall impact of the application is deemed to be moderate-to-high.

1. Significance:

Strengths

- The study will address a population in need of intervention. Chronic orofacial pain (COP) is debilitating, with current treatments being suboptimal and limiting treatment engagement for this particular population.
- The investigators have developed a plausible conceptual model supporting the association between the intervention skills and pain-related outcomes, including potential mechanisms that mediate treatment effects.

- The goals of assessing feasibility and acceptability, and making iterative changes to the treatment, are appropriate for this early stage of research in this area and align with the NIH Stage Model for Behavioral Intervention Development.
- The investigators have a clearly outlined plan regarding future steps for this research.

Weaknesses

- Concerns about the definition of the sample and other aspects of the approach reduce potential significance.

2. Investigator(s):

Strengths

- MPIs Greenberg and Vranceanu have strong evidence of scientific productivity and have expertise in psychosocial and mind-body interventions for chronic pain.
- The study team is highly productive and has developed strong research collaborations supporting the successful completion of the study.
- The co-investigators complement the study team by bringing together expertise in biostatistics, general dentistry, intervention dissemination, and web programming of the intervention.
- The MPI plan seems appropriate. The MPIs will have weekly team meetings, weekly check-ins with the RA, and a team meeting once yearly to discuss the study protocol and progress. The MPIs have a history of collaboration, thus providing support for their successful partnership in the proposed project.

Weaknesses

- None noted.

3. Innovation:

Strengths

- While the individual components of the intervention have been used in other MBSR and CBT trials, the integration of skills and concepts adapted for the targeted population is novel.
- Per the investigators, there has only been one other study using an asynchronous web-based platform for adults with COP.

Weaknesses

- Prior work has evaluated web-based platforms for intervention dissemination. The proposed project is only incrementally novel in this respect.

4. Approach:

Strengths

- The recruitment plan appears sufficient to generate the required number of participants given collaborations with existing recruitment sources (e.g., FPA has approximately 30,000 members with orofacial pain).
- Preliminary data are strong and highlight the feasibility of the proposed recruitment targets and potential efficacy of the intervention in the targeted population.

- The design of the RP3 intervention is iterative and will integrate both qualitative and quantitative data to refine the components of the intervention.
- The investigators plan to examine participant satisfaction, engagement with treatment, intervention credibility, and feasibility of recruitment, with metrics in place to determine success.

Weaknesses

- The sample is inadequately characterized. The study proposes to recruit patients with heterogeneous COP. There is no justification for this, nor are there any details regarding what types of diagnoses will be considered for inclusion or exclusion. The impact of this on patient generalizability is not discussed.
- There are no details on how patient diagnosis will be assessed and confirmed. Will diagnosis be based on self-report of symptoms?
- A justification for the intervention is that talk-free treatments provide a platform for improvement in COP given difficulties with talking. How will the investigators ensure that they recruit a sample with orofacial limitations that might benefit from the intervention? While there is a pain severity threshold considered for inclusion, there is no criteria regarding functional limitations.
- Recruitment statistics from MGH are from 2016. Given the feasibility and pilot nature of the design, recruitment is likely not going to be an issue, but an update on the statistics would have provided more accurate estimates on recruitment demographics.
- While adverse effects are likely to be modest given the nature of the intervention, more clearly defined metrics for signifying “acceptable” metrics are needed.

5. Environment:

Strengths

- The research environment at Massachusetts General Hospital is excellent and is likely to facilitate successful completion of the project.

Weaknesses

- None noted.

Study Timeline:

Strengths

- The timeline is appropriate and feasible.

Weaknesses

- None noted.

Protections for Human Subjects

Acceptable Risks and/or Adequate Protections

- Adequate protections are in place to mitigate risk.

Data and Safety Monitoring Plan (Applicable for Clinical Trials Only):

Acceptable

- The MPIs have included an acceptable DSMP, although one was not required.

Inclusion Plans

- Sex/Gender: Distribution justified scientifically.
- Race/Ethnicity: Distribution justified scientifically.
- Inclusion/Exclusion Based on Age: Distribution justified scientifically.
- Participants will be 60% women, ages 18 years and older. There is no upper age limit. This seems reasonable given the targeted population. The investigators will recruit approximately 65% White, 10% Black, 10% Asian, 5% Native Hawaiian, 10% Biracial, and 10% Hispanic in accordance with MGH estimates.

Vertebrate Animals

Not Applicable (No Vertebrate Animals)

Biohazards

Not Applicable (No Biohazards)

Resubmission

- The resubmission was responsive to the previous reviews. The investigators have addressed prior concerns by enhancing the rigor of the prior literature, addressing unequal group sizes, providing a recruitment and retention plan, and clarifying aspects of the methodology and innovation.

Budget and Period of Support

Recommend as Requested

Recommended budget modifications or possible overlap identified:

- Budget appears appropriate. No funds are requested for data management and sharing.

CRITIQUE 2

Significance: 1
Investigator(s): 1
Innovation: 3
Approach: 2
Environment: 1

Overall Impact: This proposal is a resubmission of a grant application from an MPI team that aims to adapt the Relaxation Response Resiliency Program (3RP) to a web-based intervention, Face-Forward-Web, for the unique needs of people with chronic orofacial pain. The proposal is significant by addressing chronic orofacial pain - a common and costly condition associated with substantial pain-related activity limitations and emotional distress. The investigator team is strong. The Multiple PIs, Drs. Greenberg and Vranceanu, have assembled a research team with the requisite expertise in clinical psychology, technology-enhanced mindbody-lifestyle interventions, and orofacial pain to accomplish the specific aims. The proposal is incrementally innovative. Although the intervention, Face-Forward-

Web, is a "talk-free" (asynchronous) psychosocial web-platform intervention for adults with heterogeneous chronic orofacial pain, other psychological management interventions for chronic orofacial pain already exist. The approach is strong. The researchers' preliminary studies indicate the need for an intervention for chronic orofacial pain that encompasses psychosocial aspects and uses a web-based platform. The proposed research design and methodology is based on the NIH model for behavioral intervention development and the researchers' prior web-platform development projects. Further, the researchers will ensure scientific rigor and reproducibility by using validated measures and including fidelity analysis via the user experience software. The research will occur at Massachusetts General Hospital (MGH), which houses multiple resources that will support the proposed research, including the Center for Health Outcomes and Interdisciplinary Research (CHOIR) led by Dr. Vranceanu and the Center for Telehealth. Overall, the proposed research has multiple strengths. The accomplishment of the specific aims is likely to exert a powerful influence that informs technology innovation and pain science.

1. Significance:

Strengths

- Chronic orofacial pain (COP) is common, costly, and associated with substantial pain-related activity limitations and emotional distress.
- Treatments are typically biomedical, mostly ineffective, and often involve adverse side effects, misdiagnoses, and unnecessary/painful procedures (e.g., tooth extractions, occlusal adjustment) that may lead to irreversible damage.
- The design of the intervention, Face-Forward-Web, is an adaptation of the Relaxation Response Resiliency Program (3RP) and is based on the fear avoidance framework.

Weaknesses

- None.

2. Investigator(s):

Strengths

- The Multiple PIs, Drs. Greenberg and Vranceanu, have assembled a research team with the requisite expertise in clinical psychology, technology-enhanced mindbody-lifestyle interventions, and orofacial pain to accomplish the specific aims.
- Drs. Greenberg and Vranceanu have established a productive collaboration.
- The Multiple PI Leadership Plan specifies that differences of opinion that may arise among the MPIs will be resolved through deliberation and consultation with a five-member ad hoc committee created for the purpose of resolving disagreements.

Weaknesses

- None.

3. Innovation:

Strengths

- The intervention, Face-Forward-Web, is a "talk-free" (asynchronous) psychosocial web-platform intervention for adults with heterogeneous chronic orofacial pain.

- The intervention will be tailored to the unique needs of people with chronic orofacial pain and iteratively refined during web platform optimization and feasibility testing in Aim 2

Weaknesses

- Other psychological management interventions for chronic orofacial pain already exist.

4. Approach:

Strengths

- The preliminary studies indicate the need for an intervention for chronic orofacial pain that encompasses psychosocial aspects and prefer a web-based platform over other delivery modalities.
- The proposed research design and methodology is based on the NIH model for behavioral intervention development and the study team's prior research.
- The researchers provide specific feasibility metrics that signal the web-based platform is optimized for the next development stage of a small pilot RCT and contingency plans if the feasibility benchmarks are not initially met.
- The researchers will ensure scientific rigor and reproducibility by using validated measures and including fidelity analysis via the user experience software.

Weaknesses

- For Aim 2, it is unclear whether the researchers are video- or audio-recording participants while they complete the intervention. The use of recordings while participants complete the intervention may capture additional information about the platform performance and intervention development.

5. Environment:

Strengths

- Massachusetts General Hospital (MGH) houses multiple resources that will support the proposed research, including the Center for Health Outcomes and Interdisciplinary Research (CHOIR) led by Dr. Vranceanu and the Center for Telehealth.
- The MGH Telehealth team provides full support to troubleshoot technical issues with the intervention's web platform.
- The research team will recruit from the Facial Pain Association, which has approximately 30,000 members with various types of orofacial pain.

Weaknesses

- None.

Study Timeline:

Strengths

- Study activities will be completed within the award period.

Weaknesses

- None.

Protections for Human Subjects

Acceptable Risks and/or Adequate Protections

- In the unlikely event that a participant will express a risk for self-harm or active suicidality, the MPI (Greenberg) will contact the participant and start a safety procedure (assessment, referral, emergency room visit, as needed) to ensure the safety of the participant.

Data and Safety Monitoring Plan (Applicable for Clinical Trials Only):

Acceptable

- The proposed qualitative focus groups and the feasibility open pilot study of the Face-Forward-Web intervention pose minimal risk and do not qualify as a clinical trial. Therefore, no Data and Safety Monitoring Plan (DSMB) will be appointed for this study.

Inclusion Plans

- Sex/Gender: Distribution justified scientifically.
- Race/Ethnicity: Distribution justified scientifically.
- Inclusion/Exclusion Based on Age: Distribution justified scientifically.
- The proposed distribution of participants is based on MGH's patient population (Approximately 70% are white, 12% Hispanic; 10% black; 6% Asian, 14% native Hawaiian/pacific islander, American Indian/Alaska-Native, or others).

Vertebrate Animals

Not Applicable (No Vertebrate Animals)

- Not applicable

Biohazards

Not Applicable (No Biohazards)

- Not applicable

Resubmission

- The researchers were responsive to reviewers' critique by clarifying and enhancing the review of existing literature on web-based interventions, highlighting that the adapted intervention is a "talk free" intervention, which addresses the unique communication challenges of the study population.

Resource Sharing Plans

Not Applicable (No Relevant Resources)

- The budget for each year of the award is <$500,000.

Budget and Period of Support

Recommend as Requested

Recommended budget modifications or possible overlap identified:

- Budget for Data Management and Sharing Costs is justified.

CRITIQUE 3

Significance: 2

Investigator(s): 1
Innovation: 3
Approach: 3
Environment: 1

Overall Impact: Chronic orofacial pain (COP) is an umbrella of several debilitating chronic pain conditions with ineffective biomedical treatments. This application is a resubmitted R21 aiming to adapt the relaxation response resiliency program (3RP) for "talk-free" delivery through a web-based platform. The intervention targets maladaptive functioning to improve pain and function. 3RP has been developed for other chronic pain conditions and piloted for COP in one study done by the investigators but it was never tailored to COP patients on a web-based platform. This R21 application aims to specifically develop and optimize 3RP on Face-Forward-Web platform to understand patients' treatment needs, feasibility, and acceptability. The Face-Forward Web based platform is talk-free, readily accessible, and scalable to large number of patients. The application will lead to preliminary data for a future larger application with potentially high impact on the field of treatment of COP. The project has some weaknesses, but these do not weaken the overall impact of the application.

1. Significance:

Strengths

- Psychosocial interventions for chronic pain are effective and low risk treatments and target critical aspects of chronic pain which is not reduced to simply an increase in nociception. A web-based approach tailoring such a treatment to COP where therapeutics are highly needed can potentially have significant impact on the field.

Weaknesses

- None.

2. Investigator(s):

Strengths

- The investigators have the necessary skills, background, and experience to complete this project. Dr. Greenberg and Vranceanu in psychosocial treatment of chronic pain and 3RP; Drs Shaefer, Kulich, and Cheng in orofacial pain and recruitment of COP patients; Dr Bakhshaie in biostatistics. Drs. Greenberg, Vranceanu, and Bakhshaie have published together.

Weaknesses

- None.

3. Innovation:

Strengths

- "Talk-free" delivery of psycho-social web-based intervention for COP patients; this approach is less painful, more accessible, cheaper, and multimodal.

Weaknesses

- None.

4. Approach:

Strengths

- The 3RP tailored to web-based accessible for COP is a strength.

- Talk -free delivery is a strength.
- Iterative approach to refine the Face Forward Web with deliverables is strength.

Weaknesses

- Within the resources of an R21 this application could have aimed for a larger sample size to reduce error and noise
- Relatedly, the analysis is mostly qualitative; more quantitative benchmarks would add strength and confidence in the approach.
- Preliminary data from published work in 2013 on the efficacy of 3RP in COP is a small uncontrolled trial.

5. Environment:

Strengths

- The environment at MGH is conducive to the successful completion of the project as it includes the necessary recruitment infrastructure (Facial Pain Clinic), resources for biostatistical analysis, and clinical research.

Weaknesses

- None.

Study Timeline:

Strengths

- Feasible timeline for recruitment and development of the Face Forward Web.

Weaknesses

- None noted.

Protections for Human Subjects

Acceptable Risks and/or Adequate Protections

Data and Safety Monitoring Plan (Applicable for Clinical Trials Only):

Not Applicable (No Clinical Trials)

Inclusion Plans

- Sex/Gender: Distribution justified scientifically.
- Race/Ethnicity: Distribution justified scientifically.
- Inclusion/Exclusion Based on Age: Distribution justified scientifically.
- enrolls whites, AA, and Hispanics..

Vertebrate Animals

Not Applicable (No Vertebrate Animals)

Biohazards

Not Applicable (No Biohazards)

Resubmission

- highly responsive to previous critique in terms of expanding on the literature of web-based interventions, how COP would produce better efficacy relative to CBT for example.

Budget and Period of Support

Recommend as Requested

Recommended budget modifications or possible overlap identified:

- no costs associated with data management.

THE FOLLOWING SECTIONS WERE PREPARED BY THE SCIENTIFIC REVIEW OFFICER TO SUMMARIZE THE OUTCOME OF DISCUSSIONS OF THE REVIEW COMMITTEE, OR REVIEWERS' WRITTEN CRITIQUES, ON THE FOLLOWING ISSUES:

PROTECTION OF HUMAN SUBJECTS: ACCEPTABLE

INCLUSION OF WOMEN PLAN: ACCEPTABLE

INCLUSION OF MINORITIES PLAN: ACCEPTABLE

INCLUSION ACROSS THE LIFESPAN: ACCEPTABLE

COMMITTEE BUDGET RECOMMENDATIONS: The budget was recommended as requested.

Footnotes for 1R21DE033502-01A1; PI Name: Greenberg, Jonathan

NIH has modified its policy regarding the receipt of resubmissions (amended applications).See Guide Notice NOT-OD-18-197 at https://grants.nih.gov/grants/guide/notice-files/NOT-OD-18-197.html. The impact/priority score is calculated after discussion of an application by averaging the overall scores (1-9) given by all voting reviewers on the committee and multiplying by 10. The criterion scores are submitted prior to the meeting by the individual reviewers assigned to an application, and are not discussed specifically at the review meeting or calculated into the overall impact score. Some applications also receive a percentile ranking. For details on the review process, see http://grants.nih.gov/grants/peer_review_process.htm#scoring.

MEETING ROSTER

Biobehavioral Medicine and Health Outcomes Study Section
Risk, Prevention and Health Behavior Integrated Review Group
CENTER FOR SCIENTIFIC REVIEW
BMHO
01/29/2024 - 01/30/2024

Notice of NIH Policy to All Applicants: Meeting rosters are provided for information purposes only. Applicant investigators and institutional officials must not communicate directly with study section members about an application before or after the review. Failure to observe this policy will create a serious breach of integrity in the peer review process, and may lead to actions outlined in NOT-OD-22-044 at https://grants.nih.gov/grants/guide/notice-files/NOT-OD-22-044.html, including removal of the application from immediate review.

CHAIRPERSON(S)

SCHENKER, YAEL, MD
PROFESSOR
DEPARTMENT OF MEDICINE
SECTION OF PALLIATIVE CARE AND MEDICAL ETHICS
UNIVERSITY OF PITTSBURGH
PITTSBURGH, PA 15261

MEMBERS

BARTLEY, EMILY J, PHD *
ASSISTANT PROFESSOR
COLLEGE OF DENTISTRY
UNIVERSITY OF FLORIDA
GAINESVILLE, FL 32610

CARLSON, JORDAN A, PHD *
ASSOCIATE PROFESSOR OF PEDIATRICS
UNIVERSITY OF MISSOURI-KANSAS CITY
SCHOOL OF MEDICINE
KANSAS CITY, MO 64108

CHENG, ABBY LING-LEE, MD *
ASSISTANT PROFESSOR
ORTHOPEDIC SURGERY, NEUROLOGY AND SURGERY
SCHOOL OF MEDICINE
WASHINGTON UNIVERSITY
SAINT LOUIS, MO 63108

D'ALONZO, KAREN THERESE, PHD, MSN *
ASSOCIATE PROFESSOR
DIVISION OF NURSING SCIENCE
SCHOOL OF NURSING
RUTGERS, THE STATE UNIVERSITY OF NEW JERSEY
NEWARK, NJ 07102

FAIRMAN, CIARAN, PHD *
ASSISTANT PROFESSOR
ARNOLD SCHOOL OF PUBLIC HEALTH
UNIVERSITY OF SOUTH CAROLINA
COLUMBIA, SC 29229

FLOWERS, ELENA R, PHD
ASSOCIATE PROFESSOR
DEPARTMENT OF PHYSIOLOGICAL NURSING
UNIVERSITY OF CALIFORNIA, SAN FRANCISCO
SAN FRANCISCO, CA 94143

FORTIER, MICHELLE, PHD
ASSOCIATE PROFESSOR
SUE AND BILL GROSS SCHOOL OF NURSING
UNIVERSITY OF CALIFORNIA, IRVINE
ORANGE, CA 92868

GALLO, LINDA C, PHD
PROFESSOR
DEPARTMENT OF PSYCHOLOGY
SAN DIEGO STATE UNIVERSITY
SAN DIEGO, CA 92123

GEHA, PAUL, MD *
ASSOCIATE PROFESSOR
DEPARTMENT OF PSYCHIATRY
YALE UNIVERSITY SCHOOL OF MEDICINE
NEW HAVEN, MA 06520

GOLDMAN, MYLA DENISE, MD *
PROFESSOR
DEPARTMENT OF NEUROLOGY
VIRGINIA COMMONWEALTH
UNIVERSITY SCHOOL OF MEDICINE
RICHMOND, VA 23292

GOLDSTEIN-PIEKARSKI, ANDREA, PHD
ASSISTANT PROFESSOR
DEPARTMENT OF PSYCHIATRY AND SLEEP MEDICINE
PSYCHIATRY AND BEHAVIORAL SCIENCES
SCHOOL OF MEDICINE
STANFORD UNIVERSITY
STANFORD, CA 94305

GONZALEZ, BRIAN D, PHD
ASSOCIATE MEMBER
DEPARTMENT OF HEALTH OUTCOMES AND BEHAVIOR
MOFFITT CANCER CENTER
TAMPA, FL 33647

HARPER, FELICITY, PHD
ASSOCIATE CENTER DIRECTOR AND PROFESSOR
DEPARTMENT OF ONCOLOGY
KARMANOS CANCER INSTITUTE
WAYNE STATE UNIVERSITY
DETROIT, MI 48201

HASSETT, AFTON L, PSYD
ASSOCIATE PROFESSOR
DEPARTMENT OF ANESTHESIOLOGY
CHRONIC PAIN AND FATIGUE RESEARCH CENTER
UNIVERSITY OF MICHIGAN MEDICAL SCHOOL
ANN ARBOR, MI 48106

HOYT, MICHAEL A, PHD *
PROFESSOR
CHAO FAMILY COMPREHENSIVE CANCER CENTER
DEPT. OF POPULATION HEALTH & DISEASE PREVENTION
INTERDISCIPLINARY SALIVARY BIOSCIENCE RESEARCH
UNIVERSITY OF CALIFORNIA, IRVINE
IRVINE, CA 92697

KOLACZ, JACEK, PHD *
ASSISTANT PROFESSOR
DEPARTMENT OF PSYCHIATRY AND BEHAVIORAL HEALTH
THE OHIO STATE UNIVERSITY
COLUMBUS, OH 43210

MARQUEZ, DAVID X, PHD
PROFESSOR
DEPARTMENT OF KINESIOLOGY AND NUTRITION
UNIVERSITY OF ILLINOIS AT CHICAGO
CHICAGO, IL 60612

MCDONALD, ANDREW M, MD *
ASSOCIATE PROFESSOR
DEPARTMENT OF RADIATION ONCOLOGY
UNIVERSITY OF ALABAMA AT BIRMINGHAM
BIRMINGHAM, AL 35294

MEYER, JACOB D, PHD *
ASSOCIATE PROFESSOR
DEPARTMENT OF KINESIOLOGY
COLLEGE OF HUMAN SCIENCES
IOWA STATE UNIVERSITY
AMES, IA 50011

PYATAK, ELIZABETH F, PHD
ASSOCIATE PROFESSOR
DIVISION OF OCCUPATIONAL SCIENCE
AND OCCUPATIONAL THERAPY
UNIVERSITY OF SOUTHERN CALIFORNIA
LOS ANGELES, CA 90089

RADHAKRISHNAN, KAVITA, PHD
ASSOCIATE PROFESSOR
SCHOOL OF NURSING
UNIVERSITY OF TEXAS AUSTIN
AUSTIN, TX 78712

RENN, CYNTHIA L, PHD
PROFESSOR
ORGANIZATIONAL SYSTEMS AND ADULT HEALTH
SCHOOL OF NURSING
UNIVERSITY OF MARYLAND
BALTIMORE, MD 21201

ROPER, JENEVIEVE LYNN, PHD *
DEPARTMENT OF HEALTH AND HUMAN SCIENCES
LOYOLA MARYMOUNT UNIVERSITY
LOS ANGELES, CA 90045

SUNEJA, GITA, MD *
ASSOCIATE PROFESSOR
DEPARTMENT OF RADIATION ONCOLOGY
UNIVERSITY OF UTAH SCHOOL OF MEDICINE
SALT LAKE CITY, UT 84112

SWARTZ, MARIA CHING-YI CHANG, PHD *
ASSISTANT PROFESSOR
DEPARTMENT OF PEDIATRICS –
RESEARCH DIVISION OF PEDIATRICS
THE UNIVERSITY OF TEXAS
HOUSTON, TX 77030

TAPIA, IGNACIO ESTEBAN, MD
CHIEF AND PROFESSOR
DIVISION OF PEDIATRIC PULMONOLOGY
BATCHELOR PROFESSOR OF CYSTIC FIBROSIS
AND PEDIATRIC PULMONOLOGY
UNIVERSITY OF MIAMI MILLER SCHOOL OF MEDICINE
MIAMI, FL 33136

VAN CLEAVE, JANET H, MSN, PHD *
ASSISTANT PROFESSOR
CIZIK SCHOOL OF NURSING
UNIVERSITY OF TEXAS HEALTH SCIENCE CENTER
AT HOUSTON
HOUSTON, TX 77030

WEN, KUANG-YI, PHD
ASSOCIATE PROFESSOR
DEPARTMENT OF MEDICAL ONCOLOGY
THOMAS JEFFERSON UNIVERSITY
PHILADELPHIA, PA 19107

WHITAKER, KARA MARIE, PHD *
ASSOCIATE PROFESSOR
DEPARTMENT OF HEALTH & HUMAN PHYSIOLOGY
COLLEGE OF LIBERAL ARTS AND SCIENCES
UNIVERSITY OF IOWA
IOWA CITY, IA 52242

YAGGI, HENRY KLAR, MD
ASSOCIATE PROFESSOR OF MEDICINE
DIRECTOR, YALE CENTERS FOR SLEEP MEDICINE
SCHOOL OF MEDICINE
YALE UNIVERSITY
NEW HAVEN, CT 06510

SCIENTIFIC REVIEW OFFICER

VOSVICK, MARK A, PHD
SCIENTIFIC REVIEW OFFICER
CENTER FOR SCIENTIFIC REVIEW
NATIONAL INSTITUTES OF HEALTH
BETHESDA, MD 20892

EXTRAMURAL SUPPORT ASSISTANT

AMARE, MERON ERMIAS
LEAD EXTRAMURAL SUPPORT ASSISTANT
CENTER FOR SCIENTIFIC REVIEW
NATIONAL INSTITUTES OF HEALTH
BETHESDA, MD 20892

* Temporary Member. For grant applications, temporary members may participate in the entire meeting or may review only selected applications as needed.

Consultants are required to absent themselves from the room during the review of any application if their presence would constitute or appear to constitute a conflict of interest.
